# The Role of Peroxiredoxins in the Mechanisms of Oxidative Stress in Patients After Aneurysmal Subarachnoid Hemorrhage

**DOI:** 10.3390/ijms27093796

**Published:** 2026-04-24

**Authors:** Karol Zaczkowski, Bartosz Szmyd, Małgorzata Podstawka, Anna Dębska, Natalia Koc, Rafał Wójcik, Ernest Jan Bobeff, Dariusz Jan Jaskólski, Karol Wiśniewski

**Affiliations:** 1 Department of Neurosurgery and Neurooncology, Barlicki University Hospital, Medical University of Łódź, Kopcińskiego 22, 90-153 Lodz, Poland; bartosz.szmyd@umed.lodz.pl (B.S.);; 2 Faculty of Medicine, Medical University of Gdansk, 80-210 Gdansk, Poland

**Keywords:** delayed cerebral ischemia, subarachnoid hemorrhage, peroxiredoxine, oxidative stress

## Abstract

Delayed cerebral ischemia (DCI) is a major complication of aneurysmal subarachnoid hemorrhage (aSAH), strongly associated with neurological deterioration and poor outcomes. Its pathophysiology remains incompletely understood and involves multiple interacting processes. Increasing evidence highlights the role of redox imbalance triggered by hemoglobin breakdown and the subsequent generation of reactive species, leading to vascular dysfunction, impaired nitric oxide signaling, and inflammatory activation This review aims to summarize current knowledge on redox-related mechanisms involved in DCI and to explore the potential role of the peroxiredoxin (PRDX) family in this setting. A narrative review of experimental and preclinical studies was performed, focusing on molecular pathways associated with vascular regulation, cellular injury, and antioxidant defense. Particular attention was given to the distribution and biological functions of PRDX isoforms within the central nervous system. This work addresses a topic not previously systematically discussed, the potential involvement of PRDX proteins in aSAH-related complications. By integrating available data, it provides a conceptual framework linking PRDX to mechanisms relevant for DCI. The manuscript serves as a starting point for future research, particularly translational and clinical studies in humans, which are necessary to verify the relevance of these findings and to better understand their potential clinical implications.

## 1. Introduction

Delayed cerebral ischemia (DCI) is the most common complication of aneurysmal subarachnoid hemorrhage (aSAH) [1]. Its occurrence is defined as the development of a new neurological deficit or a decrease in the level of consciousness by at least 2 points on the Glasgow Coma Scale (GCS) for at least 2 h [2]. When DCI occurs, it significantly worsens the patient’s prognosis, impairing neurological function and even leading to death. Due to its frequent occurrence and poor outcomes, methods for the effective diagnosis and prevention of DCI are being sought [3,4]. This task, however, is very difficult, as DCI is most likely multifactorial, and its pathogenesis is still debated.

Currently, much research focuses on oxidative stress (OS) as a potential overarching cause of DCI. It is directly or indirectly linked to other proposed pathomechanisms of DCI, and increasing evidence in the literature suggests that failure of antioxidant mechanisms predisposes patients to the development of DCI [5,6,7]. OS represents a cascade of reactions after the aSAH, the exact course of which has never been fully elucidated. Available data are largely limited to analyses of selected pathways. Peroxiredoxins (PRDX) are enzymes belonging to the peroxidase family, whose primary role in the organism is the reduction of hydrogen peroxide (H_2_O_2_) [8,9]. To date, six subtypes have been identified in humans. Studies conducted in animal models indicate that they play a key role in reducing H_2_O_2_, which belongs to reactive oxygen species (ROS). However, their role is not limited solely to functioning as antioxidant enzymes; they are also believed to participate in signaling processes regulating H_2_O_2_. All PRDX subtypes have been linked in some way to neurological diseases, including both ischemic and hemorrhagic stroke. However, to date, no detailed studies have been conducted on their activity in patients with aSAH. Given the complex pathomechanism of DCI, it is unlikely that PRDX play a primary role in its development. Nevertheless, in a broader context, as one of the antioxidant defense mechanisms, their dysfunction may predispose patients to its occurrence [10].

This article presents the current state of knowledge regarding PRDX and discusses their potential role in patients with aSAH based on studies conducted in animal models. We believe that to better understand the mechanisms leading to DCI, it is necessary to analyze and investigate all antioxidant mechanisms. However, little is known about PRDX and their role in aSAH, and therefore their disfunction or deficiency may be considered potential contributors to the pathogenesis of both DCI and OS.

## 2. Oxidative Stress as Delayed Cerebral Ischemia Contributor

Inflammation and oxidative stress are among the earliest mechanisms implicated in the development of DCI after aSAH. Numerous studies indicate that an intensified inflammatory response accompanied by increased oxidative stress is associated with a higher incidence of DCI and poorer clinical outcomes [11,12,13]. Following hemorrhage, erythrocytes entering the subarachnoid space undergo lysis, leading to the release of hemoglobin and its degradation products. Hemoglobin is scavenged by haptoglobin (HP), which occurs in two major isoforms, HP1-1 and HP2-2. The HP2-2 phenotype has been associated with a stronger proinflammatory response, including cytokine release, leukocyte activation, and increased expression of cell adhesion molecules (CAMs), which facilitate leukocyte infiltration into the vascular wall [14,15,16]. These processes contribute to vascular dysfunction and amplify oxidative injury within the cerebral microenvironment. Several vasoactive mediators influenced by oxidative stress are believed to play an important role in the pathogenesis of DCI. Among them, endothelin-1 (ET-1), ROS, and dysregulated nitric oxide (NO) signaling are particularly important regulators of vascular tone [17,18]. ET-1 levels typically peak between the third and fourth day after SAH and contribute to vasoconstriction partly through feedback inhibition of endothelial nitric oxide synthase (eNOS), thereby reducing NO-mediated vasodilation [19,20]. Oxidative stress can disrupt NO signaling through several mechanisms. One of the key processes is the uncoupling of eNOS, which results in reduced NO production and increased generation of superoxide. This phenomenon may occur because of ROS-mediated phosphorylation of eNOS via protein kinase C (PKC) and proline-rich tyrosine kinase 2 (PYK-2), S-glutathionylation, depletion of tetrahydrobiopterin, and oxidation of essential cofactors required for eNOS activity. Additionally, increased levels of asymmetric dimethylarginine (ADMA), an endogenous inhibitor of nitric oxide synthase, have been detected in the cerebrospinal fluid of patients after SAH and correlate with both the severity and resolution of vasospasm [21,22]. Another redox-sensitive pathway involves soluble guanylyl cyclase (sGC), a key mediator of NO-dependent vasorelaxation. Under physiological conditions, binding of NO to sGC stimulates the production of cyclic guanosine monophosphate (cGMP), leading to vascular smooth muscle relaxation. Oxidative stress may impair this signaling cascade through oxidation of critical cysteine residues and loss of the heme moiety within sGC, which decreases its sensitivity to NO and promotes vasoconstriction [23,24]. Interactions between endothelin-1 signaling and NADPH oxidase (NOX) represent an additional mechanism linking OS with vascular dysfunction. ROS generated through NOX activation enhance ET-1 expression and potentiate its vasoconstrictive effects, creating a self-amplifying cycle that further increases oxidative stress and contributes to cerebral vasospasm [25,26]. OS also affects prostanoid metabolism by shifting prostacyclin synthesis toward the formation of vasoconstrictive isoprostanes, which may additionally exacerbate vascular constriction [27,28]. Beyond luminal vascular mechanisms, OS may also act through “outside-in” signaling pathways. Perivascular adipose tissue produces a variety of vasoactive substances, including NO, ROS, and adipokines, which can modulate endothelial function from the adventitial side. This mechanism has been proposed as one potential explanation for endothelial dysfunction observed after aSAH. Experimental data also suggest that oxidative stress–induced endothelial apoptosis may contribute to the development of DCI, while preservation of endothelial integrity appears to mitigate its severity [22,23]. A further consequence of excessive ROS generation is the interaction between superoxide and NO, leading to the formation of peroxynitrite (ONOO^−^), a highly cytotoxic molecule capable of initiating lipid peroxidation and damaging both endothelial and neuronal cells [29,30]. At the same time, OS impairs endogenous antioxidant defense systems, including superoxide dismutase (SOD), glutathione-dependent pathways, and the thioredoxin system [31,32]. This imbalance between ROS production and antioxidant capacity promotes a sustained pro-oxidative environment within the injured brain. The brain relies heavily on NADPH-dependent antioxidant mechanisms, including glutathione reductase, catalase (CAT), and the thioredoxin system. These systems depend on the activity of glucose-6-phosphate dehydrogenase (G6PD), which provides the reducing equivalents required for NADPH generation. Impaired G6PD activity may therefore compromise cellular redox homeostasis, affecting lipid metabolism, nitric oxide signaling, and the detoxification of ROS [33,34]. Clinical observations support the involvement of impaired antioxidant defenses in aSAH. Reduced activity of key enzymes, such as Zn/Cu–superoxide dismutase, has been reported in both cerebrospinal fluid and plasma after aSAH, with lower levels associated with unfavorable outcomes and increased risk of DCI [35,36]. In addition, alterations in the ratio between superoxide dismutase and glutathione peroxidase (GSH-Px) have been observed, which may promote the formation of highly reactive hydroxyl radicals [37,38]. The thioredoxin system has also been implicated, as elevated plasma thioredoxin levels positively correlate with disease severity and poorer prognosis following SAH or intracerebral hemorrhage. Despite extensive experimental evidence supporting the role of OS in DCI, therapeutic strategies targeting oxidative pathways have so far produced inconsistent clinical results. Several antioxidant agents, including tirilazad mesylate, ebselen, and Nicaraven, have shown promising neuroprotective effects in experimental models but failed to demonstrate clear benefits in clinical trials. This discrepancy likely reflects the multifactorial nature of DCI, heterogeneous patient populations, and the complex dynamics of redox signaling following aSAH [37,38,39,40]. Overall, the inflammatory and oxidative cascades triggered by the initial hemorrhage are highly complex and interconnected. Rather than representing a single pathway, OS in aSAH involves a network of interacting redox reactions that affect vascular tone, cellular metabolism, and inflammatory signaling. Most available studies have focused on individual components of these pathways, while a comprehensive understanding of their interactions remains limited. One of the key reactive species generated in this context is hydrogen peroxide (H_2_O_2_). As previously mentioned, erythrocytes released from the vascular bed undergo breakdown, leading to the conversion of oxyhemoglobin to methemoglobin with the release of iron, the superoxide anion (O_2_^−^), and H_2_O_2_. Their formation is further intensified by mitochondrial dysfunction occurring after hemorrhagic injury. Free Fe^3+^ reacts with H_2_O_2_ in Fenton-type reactions, leading to the formation of highly reactive hydroxyl radicals (OH•), which induce lipid peroxidation, DNA damage, and cellular apoptosis. Moreover, H_2_O_2_ promotes the formation of ferrylhemoglobin (Fe^4+^), which initiates an autocatalytic cycle of lipid oxidation. Several therapeutic approaches aimed at limiting H_2_O_2_-induced oxidative injury have been investigated in the context of DCI. These include the use of iron chelators and attempts to introduce hydrogen-containing respiratory gas mixtures to reduce H_2_O_2_-induced OS. However, the results of these strategies have so far remained unsatisfactory. Hydrogen peroxide can be detoxified through multiple antioxidant mechanisms involving catalase, glutathione-dependent enzymes, and the thioredoxin system. Among these, peroxiredoxins constitute a major class of thiol-dependent peroxidases responsible for the reduction in H_2_O_2_ and organic peroxides in mammalian cells. Despite their central role in cellular redox regulation, the potential contribution of peroxiredoxins to the pathophysiology of aSAH and the development of delayed cerebral ischemia remains largely unexplored (Table 1).

## 3. Peroxiredoxin-1

**Figure 1:**
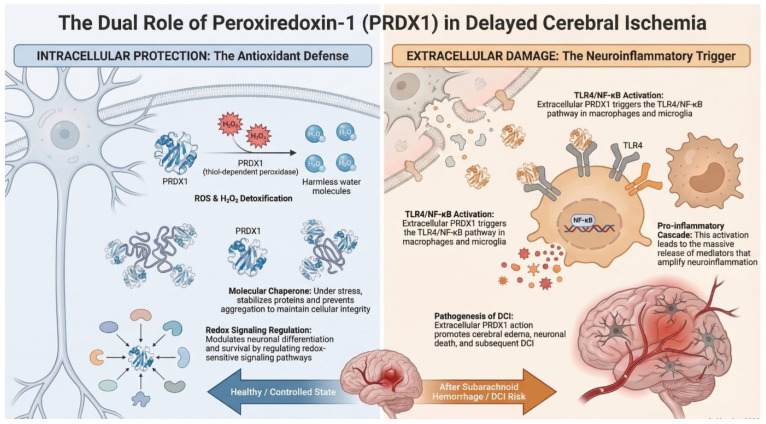
The figure illustrates the mechanisms of action of the PRDX-1 enzyme.

PRDX1 belongs to the family of thiol-dependent antioxidant enzymes that play an important role in cellular protection against oxidative stress. The protein was initially identified in studies comparing normal and RAS-transformed human mammary epithelial cells [41]. Subsequent analyses showed that PRDX1 is constitutively expressed in many human tissues, with particularly high expression observed in organs characterized by intensive cellular proliferation. Further investigations also identified PRDX1-related proteins in erythrocytes as factors capable of enhancing natural killer cell activity, suggesting that members of the peroxiredoxin family may participate both in immune regulation and in cellular responses to OS [42]. The induction of these proteins under conditions of increased oxidative burden supported their role in protecting cells from reactive oxygen species. PRDX1 primarily functions as a thiol-dependent peroxidase that reduces hydrogen peroxide and other peroxides, thereby contributing to the maintenance of intracellular redox balance. In addition to its enzymatic antioxidant activity, PRDX1 may also act as a molecular chaperone under stress conditions, helping to stabilize proteins and prevent their aggregation. Experimental studies have demonstrated that PRDX1 is expressed in differentiating motor neuron cells during spinal cord development [43]. In this context, PRDX1 regulates the activity of specific signaling proteins through redox-dependent reduction in disulfide bonds, highlighting its role not only in detoxification of ROS but also in the modulation of redox-sensitive signaling pathways. Through its interactions with the thioredoxin system, PRDX1 forms part of a broader antioxidant network responsible for controlling hydrogen peroxide levels and maintaining cellular redox homeostasis. By regulating both OS and redox signaling, PRDX1 contributes to the protection of cells from oxidative damage and supports normal cellular function. The role of peroxiredoxins as brain-protective factors has also been supported by experimental animal models, in which increased expression of PRDX1 was observed in astrocytes [44]. Experimental studies also suggest that PRDX1 may participate in inflammatory signaling after hemorrhagic brain injury. In a collagenase-induced mouse model of intracerebral hemorrhage, extracellular PRDX1 was shown to activate the TLR4/NF-κB pathway in macrophages, promoting the production of proinflammatory mediators and contributing to neuroinflammatory injury [45]. Interestingly, in the study by Richard et al., PRDX1 was identified as a promising biomarker for the onset of cerebral infarction, which may suggest that PRDX1 could also have prognostic potential in predicting DCI (Figure 1) [46]. Moreover, it has been demonstrated that induced overexpression of peroxiredoxin-1 in astrocytes and peroxiredoxin-2 in neurons attenuated OS and inhibited neuronal apoptosis following aSAH [47]. 

## 4. Peroxiredoxin-2

**Figure 2:**
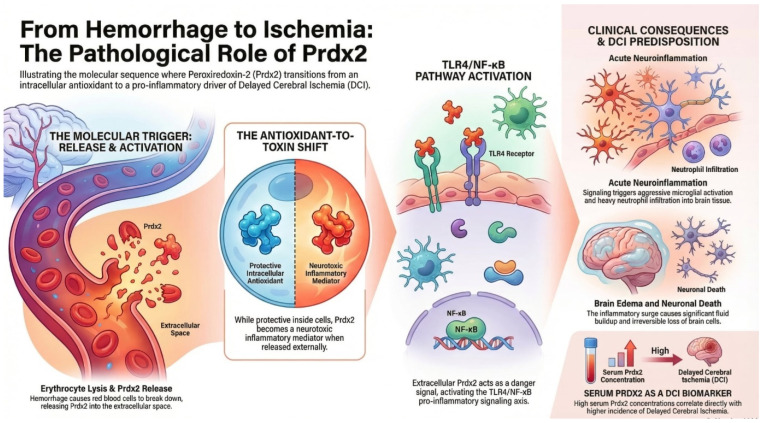
The figure illustrates the mechanisms of action of the PRDX-2 enzyme.

PRDX2 is another member of the PRDX family primarily involved in the regulation of cellular redox balance. The protein was originally identified as a natural killer cell–enhancing factor (NKEFB) during studies investigating erythrocyte-derived factors that modulate immune cell activity [42]. Subsequent research demonstrated that PRDX2 belongs to the group of thioredoxin-dependent peroxidases capable of reducing hydrogen peroxide and other ROS, thereby protecting cellular macromolecules from oxidative damage [48]. PRDX2 is widely expressed in many cell types but is particularly abundant in erythrocytes, where it constitutes one of the major antioxidant proteins responsible for maintaining redox stability. Experimental animal models have demonstrated the physiological importance of this enzyme: mice lacking PRDX2 develop hemolytic anemia associated with elevated reactive oxygen species, oxidative damage to erythrocyte proteins, and compensatory hematologic responses. These findings indicate that PRDX2 plays a crucial role in protecting red blood cells from OS. Beyond its antioxidant activity, PRDX2 has also been implicated in the regulation of intracellular signaling pathways. Studies have shown that PRDX2 can modulate platelet-derived growth factor (PDGF) signaling by limiting local peroxide accumulation, thereby preventing excessive activation of PDGF receptors and downstream proliferative signaling pathways [49]. Through such redox-dependent regulation, PRDX2 contributes to the control of cell proliferation and vascular remodeling. Interestingly, peroxiredoxins, including PRDX2, have also been shown to undergo cyclic redox oscillations with a periodicity of approximately 24 h in human erythrocytes, suggesting a potential role as conserved markers of cellular circadian rhythms [50]. These oscillations occur independently of transcriptional activity and may represent an evolutionarily conserved mechanism linking redox homeostasis with biological timekeeping. Recent studies further suggest that PRDX2 may function as a sensor of OS during DNA replication [51]. Under conditions of increased ROS, PRDX2 undergoes structural changes that influence the activity of proteins involved in replication fork progression, thereby slowing DNA synthesis and helping to prevent replication-associated genomic damage. Taken together, these observations indicate that PRDX2 plays multiple roles in cellular physiology. In addition to its classical antioxidant function in detoxifying ROS, PRDX2 participates in redox signaling, regulation of cell proliferation, and cellular stress responses, highlighting its importance in maintaining redox homeostasis in both physiological and pathological conditions. In a mouse model of intracerebral hemorrhage, PRDX2 appeared to act through the TLR4 signaling pathway, enhancing the inflammatory response and consequently leading to brain edema, microglial activation, neutrophil infiltration, neuronal death, and neurological deficits [52]. There are also reports indicating that PRDX2 may contribute to inflammation within the choroid plexus and damage to the ventricular wall, which in turn promotes the development of hydrocephalus [53]. Moreover, higher serum concentrations of PRDX2 in patients with aSAH have been associated with poorer clinical outcomes and a higher incidence of DCI (Figure 2) [54].

## 5. Peroxiredoxin-3

**Figure 3:**
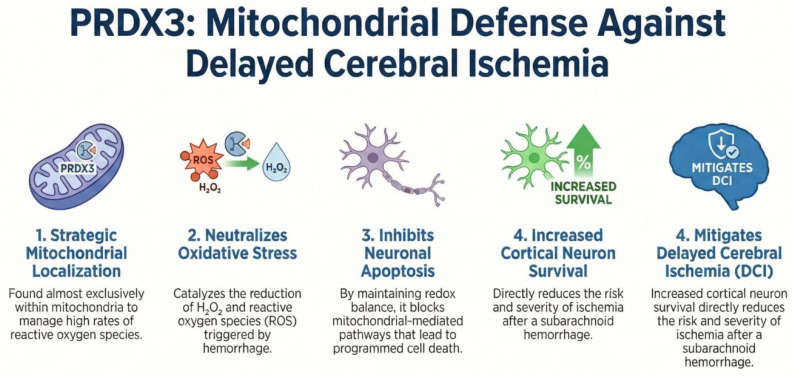
The figure illustrates the mechanisms of action of the PRDX-3 enzyme.

PRDX3 is an antioxidant enzyme that catalyzes the reduction of hydrogen peroxide and other peroxides. Its catalytic mechanism involves the oxidation of a conserved cysteine residue, which is subsequently reduced by the thioredoxin system. Thioredoxin, in turn, is regenerated by thioredoxin reductase using electrons derived from NADPH. Through this redox cycle, peroxiredoxins help maintain intracellular redox balance and protect cells against oxidative stress. Unlike several other PRDXs family members, PRDX3 is localized almost exclusively within mitochondria, where it plays a central role in detoxifying hydrogen peroxide and maintaining mitochondrial redox homeostasis due to the high rate of ROS generation during oxidative phosphorylation [55]. The protein was initially identified in studies on murine erythroleukemia cells, where it was suggested to participate in early stages of cellular differentiation [56]. Subsequent research demonstrated high sequence conservation between mammalian PRDX3 and other thiol-specific antioxidant proteins, highlighting its evolutionary and functional importance. Functional studies indicate that PRDX3 contributes not only to antioxidant defense but also to the regulation of mitochondrial integrity and cell survival [57]. It can inhibit mitochondrial cytochrome c release and attenuate apoptosis under oxidative stress. PRDX3 expression is regulated by transcription factors involved in metabolism and stress responses, including MYC and FOXO3A, underscoring its role in cellular adaptation to metabolic and oxidative challenges [58]. Genetic studies have demonstrated the clinical relevance of PRDX3. Mutations in the PRDX3 gene are associated with autosomal recessive spinocerebellar ataxia type 32 (SCAR32), a disorder marked by progressive cerebellar dysfunction. Patient-derived cells show reduced PRDX3 expression, mitochondrial dysfunction, elevated reactive oxygen species, and increased susceptibility to oxidative stress. Experimental models further confirmed that PRDX3 deficiency impairs neuronal viability and promotes neurodegeneration. In animal models, PRDX3 has been shown to exert a neuroprotective role in early brain injury after aSAH, enhancing the survival of cortical neurons and improving both short- and long-term functional outcomes [59]. Its protective mechanism appears to involve inhibition of mitochondrial-mediated neuronal death pathways. Additionally, increased sensitivity to OS has been observed in patients with defective mitochondrial PRDX3 (Figure 3) [60].

## 6. Peroxiredoxin-4

**Figure 4:**
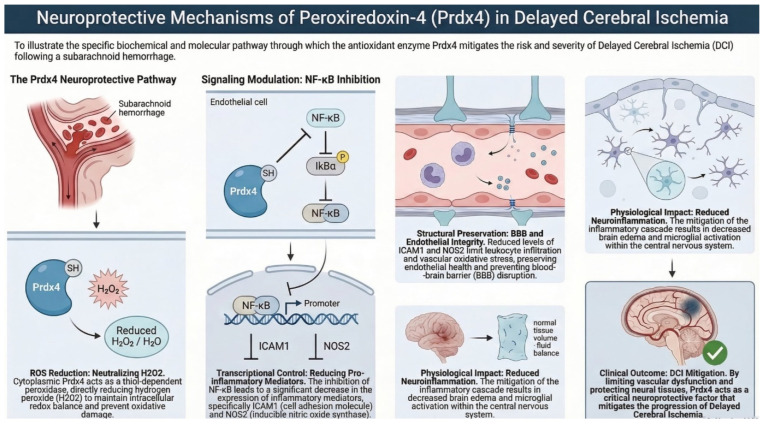
The figure illustrates the mechanisms of action of the PRDX-4 enzyme.

PRDX4 regulates intracellular redox balance and modulates redox-sensitive signaling pathways, including NF-κB activation [61]. Its catalytic activity involves reduction of hydrogen peroxide, which contributes to protection against OS and regulation of inflammatory responses. PRDX4 was originally identified in human cell lines using yeast two-hybrid screening and 5′-RACE, and was shown to contain conserved cysteine motifs characteristic of peroxiredoxins, along with an N-terminal signal sequence enabling secretion. The protein is predominantly localized in the cytoplasm but can also be secreted, and it is widely expressed in human tissues, with particularly high levels in the pancreas, liver, heart, spleen, thymus, and reproductive organs. Its expression is inducible under OS, such as exposure to H_2_O_2_, supporting its role in cellular protection. Functionally, PRDX4 has been shown to form both homo- and heterodimers with other cytoplasmic peroxiredoxins (e.g., PRDX1), modulating redox-sensitive signaling. It can influence NF-κB activation, IκBα phosphorylation, and proinflammatory mediator expression, including ICAM1 and NOS2, highlighting its role in the regulation of inflammatory pathways. In addition, PRDX4 protects key enzymes such as glutamine synthetase from oxidative inactivation and promotes cell survival under stress conditions. Animal studies have demonstrated tissue-specific roles of PRDX4 in the ovary [62], PRDX4 is highly expressed in granulosa cells, with levels increasing during follicle development and declining with age. OS induces PRDX4 expression, which correlates with protection from ROS-mediated damage and maintenance of cell proliferation. The human PRDX4 gene is located on chromosome Xp22.11. Taken together, these observations indicate that PRDX4 functions as both a cytoplasmic and secreted antioxidant, participating in redox regulation, inflammatory signaling, and protection from oxidative injury in multiple tissues. PRDX4 seems to play a critical neuroprotective role in the central nervous system by preserving endothelial integrity, limiting blood–brain barrier disruption, and reducing neuroinflammation after ischemia/reperfusion injury (Figure 4) [63].

## 7. Peroxiredoxin-5

**Figure 5:**
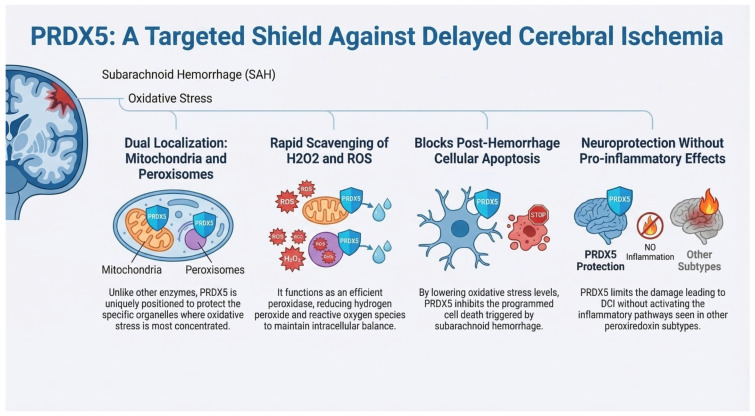
The figure illustrates the mechanisms of action of the PRDX-5 enzyme.

PRDX5 reduces hydrogen peroxide and other ROS, contributing to the maintenance of intracellular redox balance. Unlike other PRDXs, PRDX5 is uniquely localized to both mitochondria and peroxisomes, positioning it to protect against OS generated in these organelles [64]. PRDX5 was initially identified in bronchoalveolar lavage fluid and lung cDNA as a 17 kDa protein, later shown to contain three cysteine residues critical for antioxidant activity and a C-terminal peroxisomal targeting sequence [65]. Its expression is ubiquitous, with highest levels in thyroid, lung, kidney, adrenal gland, heart, and colon, and is inducible under oxidative or inflammatory stress, such as exposure to lipopolysaccharide or proinflammatory cytokines. Functionally, PRDX5 acts as an efficient scavenger of hydrogen peroxide, with antioxidant activity comparable to catalase. Studies in human fibroblasts, cartilage, and endothelial cells demonstrate that PRDX5 upregulation correlates with reduced peroxide accumulation, highlighting its protective role under both physiological and pathological conditions. Structural analyses revealed a thioredoxin-like fold, supporting its function within the thioredoxin-dependent antioxidant system. PRDX5 can be regenerated after overoxidation through sestrin-dependent mechanisms, maintaining the cellular antioxidant defense even under sustained OS. Genetically, the PRDX5 gene is located on chromosome 11q13, a region linked to atopic hypersensitivity. Overall, PRDX5 is a versatile antioxidant enzyme that protects multiple tissues from oxidative damage, supports mitochondrial and peroxisomal redox homeostasis, and participates in cellular stress responses. In animal studies, upregulation of PRDX5 was shown to reduce OS and apoptosis in rats after aSAH [66]. Interestingly, in studies conducted by Kunze et al., decreased plasma levels of PRDX5 were observed in patients with severe stroke, which may suggest that, unlike other PRDXs, it does not exert a proinflammatory effect. Moreover, the reason for the reduced PRDX5 levels in patients with severe stroke was not clarified; it is therefore unclear whether this reduction reflects impaired synthesis or a deficiency in individuals with extensive strokes, and whether it could serve as a prognostic factor (Figure 5) [67].

## 8. Peroxiredoxin-6

**Figure 6:**
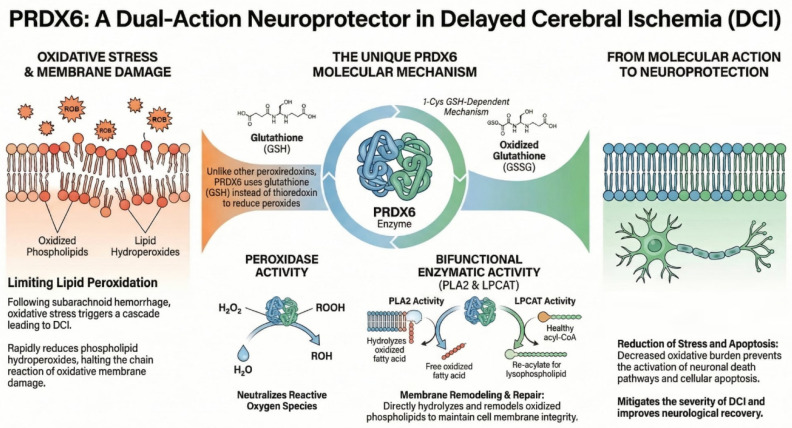
The figure illustrates the mechanisms of action of the PRDX-6 enzyme.

PRDX6 is a distinctive member of the PRDXs family, unique among mammalian peroxiredoxins in possessing only a single conserved cysteine residue and functioning through glutathione (GSH)-dependent mechanisms rather than the thioredoxin system used by PRDX1–5 [68]. PRDX6 participates in the reduction of hydrogen peroxide, short-chain hydroperoxides, and phospholipid hydroperoxides, contributing to cellular protection against oxidative stress and maintenance of redox homeostasis [69]. Unlike other peroxiredoxins, PRDX6 exhibits bifunctional enzymatic activity. In addition to its peroxidase activity, which reduces oxidized substrates, PRDX6 possesses calcium-independent phospholipase A_2_ (PLA_2_) activity and acyltransferase (LPCAT) activity, allowing it to hydrolyze and remodel oxidized phospholipids in cell membranes [70]. These multifunctional activities position PRDX6 not only as an antioxidant enzyme but also as a regulator of membrane lipid metabolism and repair following oxidative damage. PRDX6 is expressed in nearly all tissues, with particularly high levels observed in lung epithelial and endothelial cells, hepatocytes, leukocytes, and other cell types exposed to high oxidative loads. Its subcellular localization is primarily cytosolic, but PRDX6 can also be found in acidic organelles such as lysosomes and lamellar bodies, reflecting its roles in both intracellular redox defense and organelle-specific lipid processing. The peroxidase activity of PRDX6 is centered on a conserved cysteine (Cys47), which, during catalysis, is reduced by GSH bound to glutathione S-transferase, completing the enzymatic cycle. The PLA_2_ activity depends on a catalytic triad (including Ser32, His26, and Asp140) and supports hydrolysis of phospholipid sn-2 ester bonds, which facilitates membrane turnover and surfactant metabolism in the lung [71]. Through its PLA_2_ activity, PRDX6 can also influence NADPH oxidase assembly and ROS production, illustrating its dual role as both an antioxidant and a modulator of redox signaling. Functionally, PRDX6 protects cells from oxidative injury by reducing peroxidized lipids and repairing oxidative membrane damage, as evidenced in models of H_2_O_2_-induced stress where PRDX6 deficiency increases susceptibility to lipid peroxidation and cell death [72]. It also serves regulatory and signaling roles, interacting with factors involved in inflammation, cellular proliferation, and apoptosis, and its expression and activity are modulated by redox-sensitive transcriptional regulators. Thus, PRDX6 is a multifunctional antioxidant enzyme that not only detoxifies peroxides but also participates in lipid metabolism and redox signaling, enabling cells to adapt to and recover from oxidative challenges (Figure 6).

## 9. Interplay Between PRDX’s Family

**Figure 7:**
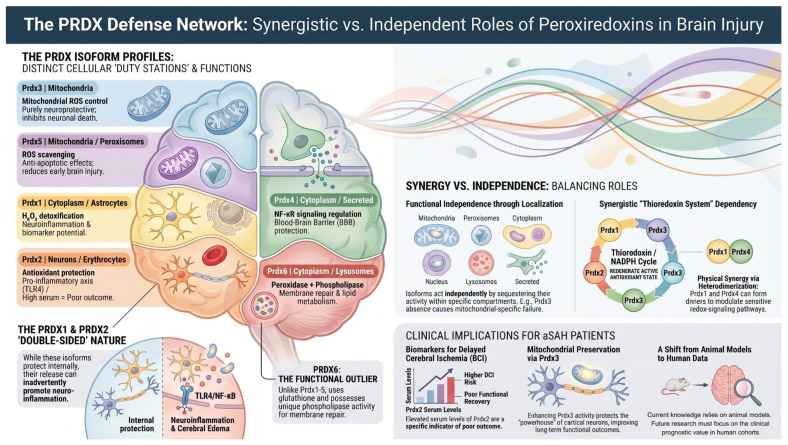
The potential role of Peroxiredoxins PRDX in the pathophysiological cascade following aneurysmal subarachnoid hemorrhage (aSAH).

The potential efficacy of the PRDX antioxidant network following aSAH relies on a strategic compartmentalization and sophisticated functional crosstalk between its members. While PRDX1 and PRDX2 provide the primary defense in the cytosol, their roles extend into the extracellular space where PRDX2, specifically released from lysed erythrocytes, acts as a Damage-Associated Molecular Pattern (DAMP) to modulate microglial inflammatory responses, thereby linking antioxidant depletion to neuroinflammation [73]. This cytosolic activity is inherently synchronized with PRDX3 and PRDX5 within the mitochondria; these isoforms form a coordinated front against metabolic oxidative stress, where PRDX3 focuses on the reduction of hydrogen peroxide while PRDX5 provides a specialized defense against peroxynitrite, preventing nitrative stress-induced neuronal apoptosis. These relationships are further characterized by functional redundancy and compensatory mechanisms that become critical during the early brain injury phase. When the classic 2-Cys peroxiredoxins (PRDX1–4) undergo hyperoxidation and subsequent inactivation due to massive ROS surges, the atypical PRDX5 and 1-Cys PRDX6, which are more resistant to such modifications, maintain residual antioxidant capacity to preserve cellular integrity [74]. Furthermore, a “floodgate” mechanism exists between PRDX1 and PRDX2, which regulate local H_2_O_2_ concentrations to facilitate the activation of redox-sensitive signaling pathways, such as the Nrf2/HO-1 axis. This activation subsequently triggers the upregulation of other family members, particularly PRDX3, creating a reinforced defensive loop against heme-mediated toxicity. A vital collaborative interaction is also observed in the protection of neuronal membranes, where the sequential action of the family is paramount; while PRDX1-5 neutralize aqueous reactive species, PRDX6 uniquely utilizes its phospholipase A2 activity to repair peroxidized phospholipids. This synergy between the prevention of damage by 2-Cys isoforms and active repair by PRDX6 is essential for preserving the blood–brain barrier and limiting the progression of secondary injury, suggesting that the clinical development of DCI is governed by the collective balance of the entire PRDX family [75]. Figure 7 presents the integrated defense network of the PRDX family, highlighting the strategic compartmentalization of isoforms within specific cellular “duty stations” to maintain redox homeostasis after aSAH. The diagram illustrates the “double-edged sword” nature of the system, where the intracellular neuroprotective and membrane-repair activities of isoforms like PRDX3 and PRDX6 are contrasted against the pro-inflammatory DAMP signaling of extracellularly released PRDX1 and PRDX2. Ultimately, the figure underscores the clinical significance of PRDX levels as biomarkers for vascular dysfunction and the development of DCI in patients.

## 10. Limitations

The primary limitation of the current state of knowledge, as discussed in this review, is the significant reliance on experimental animal models. While these models have provided invaluable insights into the basic mechanisms of PRDX and their potential role in neuroprotection or neuroinflammation after aSAH, they do not fully replicate the complex physiological and pathological environment of the human brain. Consequently, there is a distinct lack of comprehensive clinical data evaluating the specific activity and prognostic value of various PRDX isoforms directly in human patients following aSAH. Furthermore, most available human studies focus on a limited number of biomarkers or isolated redox pathways, which may not capture the multifactorial and interconnected nature of DCI development. Recognizing these gaps, the authors of this article plan to initiate fundamental research aimed at systematically expanding clinical data in human cohorts. These future studies will focus on bridging the transition from animal models to clinical practice, with the goal of better understanding the role of peroxiredoxins in patients after hemorrhage and identifying more effective, translationally relevant therapeutic targets.

## 11. Conclusions

The PRDX family serves as a critical yet dual-natured defense network in aSAH patophysiology, balancing mitochondrial neuroprotection (PRDX3, 5, 6) against pro-inflammatory DAMP signaling (PRDX1, 2). By regulating hydrogen peroxide through the thioredoxin system, these enzymes maintain blood–brain barrier integrity and modulate the oxidative-inflammatory cascade. Elevated serum levels of specific isoforms, particularly PRDX2, function as significant prognostic biomarkers for delayed cerebral ischemia (DCI) and poor clinical outcomes. Furthermore, unique mechanisms like the membrane-repair activity of PRDX6 present novel but at this point, only potential—therapeutic targets for mitigating secondary brain injury. Moving forward, large-scale human clinical studies are essential to translate these experimental insights into improved management strategies for aSAH patients.

## Figures and Tables

**Table 1:** Represents data describing PRDX enzymes.

Peroxiredoxin	Localization	Main Function	Role in CNS
PRDX1	Cytoplasm, Astrocytes	H_2_O_2_ detoxification, redox signaling	neuroinflammation
PRDX2	Erythrocytes; Neurons	antioxidant protection	neuroinflammation
PRDX3	Mitochondria	mitochondrial ROS control	neuroprotection
PRDX4	Cytoplasm/Secreted	regulation of NF-κB signaling	BBB protection
PRDX5	Mitochondria, Peroxisomes	ROS detoxification	anti-apoptotic effects
PRDX6	Cytoplasm, Lysosomes	peroxidase + phospholipase activity	membrane repair

## Data Availability

No new data were created or analyzed in this study. Data sharing is not applicable.
